# Serological and entomological survey of canine leishmaniasis in Lampedusa island, Italy

**DOI:** 10.1186/s12917-018-1606-x

**Published:** 2018-09-19

**Authors:** Valentina Foglia Manzillo, Manuela Gizzarelli, Fabrizio Vitale, Serena Montagnaro, Alessandra Torina, Salvo Sotera, Gaetano Oliva

**Affiliations:** 1 Department of Veterinary Medicine and Animal Production, University of Naples Federico II, 80137 Naples, Italy; 2 Istituto Zooprofilattico Sperimentale della Sicilia – CreNaL, 90129 Palermo, Italy; 3 DVM, Lampedusa, Italy

**Keywords:** Epidemiology, Canine leishmaniosis, Sand flies, Zoonosis, Lampedusa island

## Abstract

**Background:**

During last decade Lampedusa island (Italy) has been interested by a deep social change caused by the massive arrival of migrants from north Africa. The goal of this study was to evaluate current CanL burden and risk factors for Visceral Leishmaniosis (VL) on Lampedusa, actually based on very few data obtained in a previous study performed fifteen years ago. Two hundred and forty-two dogs were enrolled for the detection of *Leishmania infantum* infection by serology. In addition, an entomological investigation was performed to confirm the presence of *Leishmania*-vectors.

**Results:**

Seroprevalence was of 54.13%. 223 sand flies specimens were collected. Among them, 4 species were identified: *Phlebotomus perniciosus*, *P. papatasi*, *P. neglectus, Sergentomia minuta, with P. perniciosus* the most abundant (67.7%; *p* < 0.01).

**Conclusion:**

The high proportion of seropositive dogs together with the presence of the most competent vector for *L. infantum*, *P. perniciosus*, demonstrate that *L. infantum* abundantly circulates in the island and may constitute a risk for people, particularly for hosted migrants.

## Background

Zoonotic Visceral Leishmaniosis (ZVL) is an important zoonotic disease which is associated with the long history of companionship between dogs and humans. ZVL, caused by *Leishmania infantum* parasites is transmitted by phlebotomine sand flies vectors. Despite provoking a limited number of overt human clinical cases – in comparison with global leishmaniosis figures – *L. infantum* represents a latent public health threat in the European Union (EU) because studies performed in several endemic foci have disclosed a high prevalence of asymptomatic parasite carriers [1]. Based on recent data, Canine Leishmaniosis (CanL) affects approximately 2.5 million dogs in Mediterranean and peri-Mediterranean areas each year [2]. CanL is characterized by different prevalence rates in reason of the geographical climate condition that allow or not the presence and abundance of sand fly vectors. *L. infantum* causes about 700 autochthonous cases of human VL each year in Mediterranean basin. The average seroprevalence in domestic dogs is less than 25% [3, 4]. Sporadic human cases of VL and cutaneous leishmaniasis (CL) caused by *L. infantum* have been reported from Lampedusa. CanL is known to occur in the island, but studies on infection prevalence are limited to one study performed fifteen years ago [5]. In that trial, a seroprevalence of 39,1% was found in stray dogs housed in an animal shelter. Three species of sand flies were identified to be present on the island, *Phlebotomus papatasi* was the most prevalent (88,8%). *P. perniciosus* (9,7%) and *Sergentomya minuta* (1,5%) were also found. During the last decade Lampedusa has experienced massive migration from north Africa. These refugers are sheltered temporarily in an emergency center near the one village, before moving to other European locations. The dog’s population has also changed due to closure of animal shelter and adoption of dogs by tourists visiting the island during the summer. At present, more than 95% of the dog are owned. Based on these dramatic shifts in both the human and canine population the goal of this study was to evaluate current CanL burden and risk factors for VL on Lampedusa.

## Methods

### Study site

The present study was carried out in Lampedusa, a small island (20,2 km^2^) in the Italian Pelagie archipelago (35°30′56″N 12°34′23″E). This archipelago is the southernmost territory of Italy (205 km from Sicily) and the nearest to North Africa (113 km). The island has calcareous soil and is characterized by the absence of forest trees, because of intense deforestation at the beginning of the last century. Compared with other places sited at the same latitude, Lampedusa has a mild climate due to consistent winds, that are strong enough to prevent normal development of vegetation. The maximum altitude is 113 m; rivers are not present and water sources are extremely scarce. During the period from April to October temperature can reach up to 30 °C (www.il meteo.it/archiviostorico). Lampedusa has a population of 6000 inhabitants; the most of them concentrated in a small village situated in the southeast of the island. Dogs largely represent the most abundant domestic animals present on the island; less than 1000 dogs have been estimated. A few small goat and sheep farms are in the central part of the island.

### Study design

The study was designed to investigate serological, clinical and parasitological findings from autochthonous dogs living on Lampedusa and to study the phlebotomine distribution on the island, to obtain useful information about the possible risk for human population. Two hundred and forty-two dogs were enrolled between mid-November 2016 to early April 2017, a period with no adult sand flies. All dogs were owned. The owners were selected among a larger group participating to a free campaign to get information on CanL prevention, carried out during routinary veterinary clinical activity. Their dogs were enrolled after the signature of an informed consent. Dogs were of different sex, breed and age. The large majority of dogs enrolled in the study were born on the island, all the others were imported there when puppies. Each dog was submitted to clinical examination and blood sample. Clinical assessment was performed by accurate inspection of dogs for the presence of any physical signs attributable to *Leishmania* infection. All data were recorded on a clinical form that included, signaling, history, particularly including the use repellents, and clinical signs, classified in the following categories: 1) Systemic signs (S), including weight loss, muscular atrophy, lethargy and pale mucous membranes; 2) Reticulo-endothelial signs (RE), including enlargement of palpable lymph nodes such as submandibular, prescapular or popliteal nodes, and splenic enlargement as determined by palpation; 3) Cutaneous signs (C), including nodules/ulcers, onychogryphosis, dry exfoliative dermatitis and alopecia; 4) Ocular signs (O), including blepharitis, keratoconjunctivitis and uveitis [6]. The collection of biological samples was performed in accordance with the national guidelines for animal welfare. Ten milliliters of blood were obtained from the jugular vein and was centrifugated to take serum for serology, then stored − 20 °C. Dogs showing clinical signs suggestive of CanL were submitted to lymph node sampling to perform qPCR useful for therapeutic follow up. Lymph node aspiration was performed from popliteal lymph nodes using fine needle aspiration technique [7]. For this purpose owners signed a different informed consent.

### Serology

The presence of anti-*Leishmania* antibodies was assessed by an indirect immunofluorescence antibody test (IFAT) in conformity of recommendation of OIE [8] using MHOM/TN/80/IPT1 as a whole parasite antigen preparation fixed on multispot slides (Bio Merieux Spa, Florence, Italy) and a fluorescent labeled anti-canine gamma globulin (Sigma Aldrich, Milan, Italy) as conjugate. Positive sera were serially diluted and tested to establish the maximum reaction titre, starting from dilution at concentration of 1:40. Positive and negative controls were included in each slide. A dog was defined as infected if positive to quantitative serology at cut-off 1:80, according to Italian National Reference Centre for Leishmaniasis (C. Re.Na.L - Istituto Zooprofilattico Palermo, Italy).

### qPCR

DNA was extracted from lymph node aspirate samples using a Invitrogen PureLink Genomic DNA Mini Kit according to manufacturer’s instructions. The PCR test was targeted to a 68 bp fragment inside the constant region of kinetoplast DNA (kDNA) (NCBI accession number AF291093) as previously described [9]. The primer sequences were: QLK2-U 5′-GGCGTTCTGCGAAAACCG-3′; QLK2-D5′-AAAATGGCATTTTCGGGCC-3′; probe: 5′-TGGGTGCAGAAATCCCGTTCA-3′ 5′FAM with 3′ BHQ labelled. Each amplification was performed in duplicate, in 20 μl reaction mixture containing 1× TaqMan Universal Master Mix (Applied Biosystem), 20 pmol/μl primers and 10 pmol/μl of probe (Qleish 2), 1 × EXO IPC Mix, 1 × EXO IPC DNA. The thermal cycling conditions were: incubation for 2′ at 50 °C for uracil-N-glycosylase activity. This step was followed by a 10′ denaturation at 95 °C and 45 cycles at 95 °C for 15″ and 60 °C for 1′ each. Results were expressed as a parasite count for ml calibrated to a standard curve. Standard curve DNA was extracted from cultured IPT1 MON1, obtained from the collection C.Re.Na.L, 1 × 10^9^ cells/ml and homogenized in 1 ml of lysis mix, (1% Tween 20, 1% Non idet P-40, e 20% Chelex). Then decimal serial dilutions of the stock solution were performed to obtain the points of the curve ranging from the DNA equivalent of 1 × 10^6^ cells to 1 cell/ml.

### Entomological findings

In addition, a phlebotomine sand flies survey was performed to confirm the presence of *Leishmania*-competent vectors and to evaluate possible variations in their distribution in urban/periurban and rural areas of the island. Sticky traps and CDC miniature light traps (Hausherr’s Machine Works, Toms River, NJ, USA) were used in five urban (*n* = 2 traps), periurban (*n* = 1 trap) and rural (n = 2 traps) sites of the island during 2 weeks of sand flies collection (first week: middle June, second week: middle July 2016). Urban sites consisted of 2 dog’s owner private homes that housing two and four dogs respectively: the periurban site was a house with courtyard housing six dogs. The rural sites consisted of two small farms where goats and sheep were bred. Sticky traps were positioned in the same locations. CDC traps (one at each site) were suspended at approximately 1.5 m above the ground and were operated from 1 h before sunset until 1 h after sunrise. The light traps were retrieved each morning and the collected phlebotomines stored in alcohol prior to identification following standard taxonomic keys. Molecular method was used for the confirmation and differentiation of sand fly species including polymerase chain reaction (PCR) of the ribosomal ITS2 region PCR as PCR-RFLP were used for genome and species confirmation [10, 11]. As there is not a clear urban vs. periurban area, these areas as entomological results were combined.

### Statistical methods

Sample size was determined [12] with the following information: expected canine prevalence of *L. infantum* (30%) based on the average prevalence of high endemic area for SOUTH ITALY [13], confidence interval (95%) and desired absolute precision (7%). *Leishmania* seroprevalence in dogs, according to the various characteristics (gender, age, and the use of ectoparasiticides) was calculated with an associated 95% confidence interval (CI). The apparent prevalence of *L. infantum* infection was calculated as the number of serologically positive dogs among the total number of dogs tested.

The true prevalence TP was estimated using standard methods [12]:

$$ \mathrm{TP}=\left(\mathrm{AP}+\mathrm{Sp}-1\right)/\left(\mathrm{Se}+\mathrm{Sp}-1\right) $$

Where

AP is the apparent prevalence;

Se is the sensitivity of the test;

Sp is the specificity of the test.

IFAT test has a sensitivity of 96% and specificity of 98% [8].

Differences in prevalence between these various groups were assessed by the two-sided chi-square *t* test. A *P* value < 0.05 was considered statistically significant.

The variables were then applied to binary logistic models to find Odds ratios for factors associated with the seroprevalence.

The sand flies species distribution according to gender and living area was calculated with an associated 95% confidence interval (CI).

The statistical analyses were performed using MedCalc software (Frank Shoonjans, V.7.2.1.0) and SPSS, version 13.0 for Windows.

## Results

A total of 131/242 samples (54,13%) resulted positive to IFAT. Twenty-three seropositive dogs (13.4%) had clinical signs; 19 resulted positive on q-PCR. Based on the classification of clinical signs, the most frequent were RE (52.6%), particularly lymph node enlargement, S (42.1%), as weight loss and C (36.8%), mainly dry exfoliative dermatitis. Statistical analysis was performed only in dogs for which complete anamnestic data were recorded (n. 176; positive dogs n. 116). No difference between positive and negative dogs was assessed in reason of the age and the use of ectoparasiticides (Table 1). 223 sand flies specimens were collected; 214 have been used for species identification. Among them, 4 species were identified: *P. perniciosus*, *P. papatasi*, *P. neglectus, S. minuta. P. perniciosus* was the most abundant (67.7%; *p* < 0.01), followed by *S. minuta* (28.5%) and *P. papatasi* (3.3%). Only one sand fly was identified as *P. neglectus* (0,5%). Phlebotomines were most present in rural area (71.96%; *p* < 0.0001), where female insects resulted prevalent (76%; *p* < 0.05). Sand flies distribution are summarized in Table 2.

**Table 1:** Influence of age and ectoparasiticide use on *Leishmania* positive dogs

Factor	*n*	%	Standard Error %	95%CI	Chi-square	*P*	OR	95%CI
Total	116	65.90	±7.00	58.9 – 72.9	-	-	-	-
Age								
< 5	79	44.80	± 7.3	37.40 - 52.13	1.563	0.2112	0.5906	0.2852 – 1.2228
> 5	37	21.01	± 6.02	14.99 – 27.03				
Anti-ectoparasite drugs								
yes	66	37.50	± 7.15	30.35 - 44.65	0.017	0.8955	1.0094	0.5381 - 1.8936
no	50	28.40	± 6.63	21.77 – 35.03

**Table 2:** Phlebotomine sand flies distribution on Lampedusa island

Area	Species	*n*	%
Urban/periurban Area	*Phlebotomus perniciosus*	52	86.6
	*Sergentomia minuta*	7	11.6
	*Phlebotomus papatasi*	1	1.6
	*Phlebotomus neglectus*	0	0
Rural	*Phlebotomus perniciosus*	93	60.38
	*Sergentomia minuta*	54	35.06
	*Phlebotomus papatasi*	6	3.9
	*Phlebotomus neglectus*	1	0.64

## Discussion

The main risk factors for ZVL in people is CanL prevalence in that area. CanL represents a severe problem among dogs of South Italy, the median 30 years prevalence calculated from 377 canine serosurveys performed in Italy dogs was 18% (range of 11–21%) [4] . More than 1/3 of Italian municipalities were endemic for CanL [14]. On the island of Lampedusa more than 50% of dogs tested were seropositive. In this community, most dogs share the same circumscribed environment as their owners, and live in urban/periurban areas. CanL in Lampedusa should be very stable, because most dogs are autochthonous and the young/middle age did not result a risk factor for the development of the infection. Most owners of infected dogs declared that they applied insecticide drugs. However, it was not possible to have detailed information about their confidence with the use of these compounds nor regarding the composition of the applied substances. This bias could justify the absence of statistical differences between positive dogs using or not insecticide drugs. The clinical signs detected on positive dogs did not differ from those usually described for CanL, and were all related to chronic form of the disease, characterized by high IFAT titer. In addition, many owners of sick dogs declared that they do not treat with specific anti-*Leishmania* drugs, or that the dogs after the first treatment were not submitted to a regular follow up. The existence of sick not treated dogs could also influence the high circulation of the parasite on the island. Four different species of sand flies were recorded, two of which are considered competent vectors of *L. infantum*. In contrast to a previous study [5] *P. papatasi* was not abundant in the trapped specimens. The difference could be due to the different way of trapping used in the two studies, the first by using mainly direct mouth aspirators near kennel or inside hen-houses, the second with the use of light traps that are less attractive for this phlebotomine species. The presence of this sand fly on the island should be considered not dangerous for people and dogs because its specificity to transmit *Leishmania major*. This parasite causes CL and is endemic in several African countries, and thus potentially imported by migrants; a risk of local transmission is however limited because of the absence on the island of the natural reservoir hosts (rodents of the Gerbillidae family). This study represents the first description of *P. neglectus* on the island, one of the four *L. infantum* vectors present in Italy. The significance of the presence of this sand fly species mainly described on the Adriatic coast of Italy and in Balkan area needs more evidences and studies. Our results show that *P. pernicious* the most competent vector for *L. infantum,* abundantly circulates both in the urban center and in the peripheral parts of the island. The high presence of this sand fly species amplifies the risk for human population, both resident and tourists. The number of cases acquired on Lampedusa is difficult to establish due to the transient tourist and refugee populations and the long incubation period of ZVL. Some tourists may develop the infection months or years after their trip on the island, with no many possibilities to distinguish this event from other potential insect-contact happened in other areas of Europe. Resident population has frequent business trips in Sicily that is considered endemic too. To complicate the risk analysis is that on the island is only present an emergency hospital, so people suffering from chronic diseases need to go to Sicily or to other Italian regions for medical care. A particular sanitary risk is represented by the very large proportion of migrants continuously present on the island. It is well known that due to the large number of landings they form an overpopulation that stays on the island for many weeks. Most of them arrive on the island during the summer, the period of maximum intensity of the vectors, and their large majority, first children and women, should be considered immune-depressed by a very long trip at limit of survival condition. What role could play the parasite *Leishmania* in these immune-imbalanced hosts should be better investigated, even considering the scarce information on their future life-condition all around the Europe. In it cannot be excluded their role as source of other *Leishmania* species, such as the anthroponotic cutaneous form due to *L. tropica* [15].

## Conclusion

Our findings confirm the very high CanL seroprevalence on Lampedusa island. The high proportion of seropositive dogs and the presence of competent vectors demonstrate that *L. infantum* abundantly circulates in the island and may constitute a severe risk for people, living or hosting there. Sanitary measures should be considered by using large scale application of sand flies repellents and with the regular use of specific anti-*Leishmania* vaccine and drug treatment of sick dogs to limit their role of reservoir.

## References

[1] Gradoni L (2013). Epidemiological surveillance of leishmaniasis in the European Union: operational and research challenges. Euro Surveill.

[2] Moreno J, Alvar J (2002). Canine leishmaniasis: epidemiological risk and the experimental model. Trends Parasitol.

[3] Dujardin JC, Campino L, Cañavate C, Dedet JP, Gradoni L, Soteriadou K (2008). Spread of vector-borne diseases and neglect of Leishmaniasis. Europe Emerg Infect Dis.

[4] Franco AO, Davies CR, Mylne A, Dedet JP, Gállego M, Ballart C (2011). Predicting the distribution of canine leishmaniasis in western Europe based on environmental variables. Parasitology.

[5] Romagnoli P (2002). Macr’ G, Gradono L, maroli M. Serological survey on canine leishmaniasis and first record of phlebotomine sand flies in the southest Italian focus of leishmaniasis: Lampedusa island, Sicily. Parassitologia.

[6] Foglia Manzillo Valentina, Di Muccio Trentina, Cappiello Sivia, Scalone Aldo, Paparcone Rosa, Fiorentino Eleonora, Gizzarelli Manuela, Gramiccia Marina, Gradoni Luigi, Oliva Gaetano (2013). Prospective Study on the Incidence and Progression of Clinical Signs in Naïve Dogs Naturally Infected by Leishmania infantum. PLoS Neglected Tropical Diseases.

[7] Moreira MA, Luvizotto MC, Garcia JF, Corbett CE, Laurenti MD (2007). Comparison of parasitological, immunological and molecular methods for the diagnosis of leishmaniasis in dogs with different clinical signs. Vet Parasitol.

[8] Office international des epizooties (O.I.E.). Manual of diagnostic tests and vaccines for terrestrial animals. 2014 2(1)11.

[9] Reale S, Lupo T, Migliazzo A, Di Mauro C, Ciprì V, Calderone S (2010). Multilocus microsatellite polymorphism analysis to characterize Leishmania infantum strains isolated in Sicily. Transbound Emerg Dis.

[10] Khalid N, Elnaiem D, Aboud M, Al Rabba F, Tripet F (2010). Morphometric and molecular differentiation of Phlebotomus (Phlebotomus) sandflies. Med Vet Entomol.

[11] Latrofa MS, Annoscia G, Dantas-Torres F, Traversa D, Otranto D (2012). Towards a rapid molecular identification of the common phlebotomine sand flies in the Mediterranean region. Vet Parasitol.

[12] Veterinary Epidemiology, Fourth Edition. © 2018 John Wiley & Sons Ltd. Published 2018 by John Wiley & Sons Ltd. Companion website: www.wiley.com/go/veterinaryepidemiology.

[13] Gramiccia M (2011). Recent advances in leishmaniosis in pet animals: epidemiology, diagnostics and anti-vectorial prophylaxis. Vet Parasitol.

[14] Gramiccia M, Scalone A, Di Muccio T, Orsini S, Fiorentino E, Gradoni L (2013). The burden of visceral leishmaniasis in Italy from 1982 to 2012: a retrospective analysis of the multi-annual epidemic that occurred from 1989 to 2009. Euro Surveill.

[15] Di Muccio Trentina, Scalone Aldo, Bruno Antonella, Marangi Massimo, Grande Romualdo, Armignacco Orlando, Gradoni Luigi, Gramiccia Marina (2015). Epidemiology of Imported Leishmaniasis in Italy: Implications for a European Endemic Country. PLOS ONE.

